# Does repeated pleural culture increase the diagnostic yield of *Mycobacterium tuberculosis* from tuberculous pleural effusion in HIV-negative individuals?

**DOI:** 10.1371/journal.pone.0181798

**Published:** 2017-07-27

**Authors:** Yousang Ko, Jinkyung Song, Suh-Young Lee, Jin-Wook Moon, Eun-Kyung Mo, Ji Young Park, Joo-Hee Kim, Sunghoon Park, Yong Il Hwang, Seung Hun Jang, Byung Woo Jhun, Yun Su Sim, Tae Rim Shin, Dong-Gyu Kim, Ji Young Hong, Chang Youl Lee, Myung Goo Lee, Cheol-Hong Kim, In Gyu Hyun, Yong Bum Park

**Affiliations:** 1 Division of Pulmonary, Allergy and Critical Care Medicine, Department of Internal Medicine, Kangdong Sacred Heart Hospital, Hallym University College of Medicine, Seoul, Republic of Korea; 2 Lung Research Institute of Hallym University College of Medicine, Chuncheon, Republic of Korea; 3 Division of Pulmonary, Allergy and Critical Care Medicine, Hallym University Sacred Heart Hospital, Anyang, Republic of Korea; 4 Division of Pulmonary, Allergy and Critical Care Medicine, Kangnam Sacred Heart Hospital, Seoul, Republic of Korea; 5 Division of Pulmonary, Allergy and Critical Care Medicine, Chuncheon Sacred Heart Hospital, Chuncheon, Republic of Korea; 6 Division of Pulmonary, Allergy and Critical Care Medicine, Dongtan Sacred Heart Hospital, Hwaseong, Republic of Korea; Institut de Pharmacologie et de Biologie Structurale, FRANCE

## Abstract

**Background:**

Despite recent advances in methods for culturing *Mycobacterium tuberculosis* (MTB), the diagnostic yield of tuberculous pleural effusion (TBPE) remains unsatisfactory. However, unlike repeated sputum cultures of pulmonary tuberculosis, little is known about the role of repeated pleural cultures. We examined whether repeated pleural cultures are associated with increased MTB yield from TBPE.

**Methods:**

A multicenter, retrospective cohort study was performed from January 2012 to December 2015 in South Korea. Patients were categorized into two groups: single- or repeated-culture groups. The diagnostic yield of MTB and clinical, radiological, and pleural fluid characteristics were evaluated.

**Results:**

Among the 329 patients with TBPE, 77 (23.4%) had repeated cultures and 252 (76.5%) had a single culture. Pleural culture was performed twice in all 77 patients in the repeated-culture group at a 1-day interval (inter-quartile range, 1.0–2.0). In the repeated-culture group, the yield of MTB from the first culture was 31.2%, which was similar to that in the single-culture group (31.2% vs. 29.8%, P = 0.887). However, the yield of MTB from the second culture (10/77, 13.0%) was more than that from the first. These results may be attributable to the insufficient immune clearance for MTB invasion into the pleural space between the first and second cultures. Over time, the yield of the second cultures decreased from 17.4% to 6.7% and then 6.3%. Finally, the overall yield of MTB in the repeated- and single-culture groups was 44.2% and 29.8% respectively (P < 0.001).

**Conclusions:**

The results showed that repeated pleural cultures increased MTB yield from TBPE in human immunodeficiency virus-negative individuals. Furthermore, repeated cultures may increase yield when carried out for two consecutive days.

## Introduction

Tuberculosis (TB) remains a major health problem worldwide [1]. Although the WHO global TB report showed that the global incidence of pulmonary TB (PTB) has reduced over time, the incidence of extrapulmonary-TB (EPTB) has increased to approximately 17% of all new and relapse cases of TB [2, 3]. Among the different types of emerging EPTB, tuberculous pleural effusion (TBPE) is one of the most common forms [4–6].

TBPE is definitively diagnosed by detection of *Mycobacterium tuberculosis* (MTB) in the pleural fluid or a biopsy specimen [5, 7]. In many cases, detection is considered difficult because of the paucibacillary characteristics of TBPE [6]. TB-PCR involving the Xpert MTB/RIF assay can be used to diagnose TBPE with high specificity, but this assay is limited by poor sensitivity [5, 7, 8]. Thus, in TB endemic areas, many cases of TBPE are clinically diagnosed based on the lymphocytic-predominant exudate and high adenosine deaminase (ADA) level in the pleural fluid [5, 6]. However, it is necessary to culture MTB from TBPE to enable clinicians to tailor the therapy, based on the drug-sensitivity test (DST). Moreover, EPTB can be caused by drug-resistant (DR) MTB, which shows a similar DR rate as PTB [9]. Recent advances in culturing methods, such as liquid media and microscopic-observation drug-susceptibility (MODS) culture, have led to an improvement in diagnostic ability. These culture methods are used to isolate MTB from clinical specimens, including pleural fluid [5, 10–12]. However, the diagnostic yield of MTB from TBPE patients is low in clinical practice [10, 12–14].

Conventionally, repeated sputum exams using consecutive specimens have been recommended to improve the efficiency of MTB detection in PTB [15–17], but there are no recommendations or policies for a similar situation in TBPE, although this process is more important for TBPE cultures without positive MTB from a respiratory specimen. No studies have examined the diagnostic efficacy of repeated pleural cultures for MTB.

This study was conducted to evaluate whether repeating pleural cultures for MTB improves the detection rate of MTB in TBPE, which has not been previously examined in clinical studies. In addition, we compared the differences between cases that were either culture-positive in the first and/or second pleural fluid specimen to identify any discriminating clinical, radiological, and pleural fluid factors, providing useful information to allow the clinician to determine which TBPE patients could be considered candidates for additional pleural cultures for MTB detection.

## Methods

### Study population and design

We performed a retrospective review of patients diagnosed with TBPE in 4 hospitals of Hallym University Medical Center with >600 beds in the Republic of Korea, an area of intermediate TB burden with a prevalence rate of 143/100,000 persons in 2013 [2]. Medical records between January 2012 and December 2015 were obtained.

During the study period, all consecutive patients aged >18 years who had been diagnosed with TBPE were screened. The exclusion criteria were patients: 1) who had received anti-TB treatment before the first pleural culture (this can affect the cultivation rate of TBPE with paucibacillary characteristics); 2) who had TBPE combined with microbiologically confirmed PTB (this was defined as culture-positive MTB in respiratory specimens, despite no radiological evidence of PTB). These cases were excluded for two reasons. First, the original aim of this study was to develop a new method for improving culture yield for MTB in TBPE. Second, the revised WHO guidelines recommend that combined PTB and TBPE should be classified as PTB [18].); 3) with a history of previously treated TB (which may confuse the presence of combined PTB or loculated features of TBPE); 4) with positive acid-fast bacilli (AFB) smear for TBPE, because these cases do not typically require repeated pleural culture for MTB, as MTB is easily isolated and not the subject of our study. Enrolled cases were classified into 2 groups according to the number of pleural cultures for MTB: single vs. repeated pleural culture group.

The protocol for this study was approved by the Institutional Review Board of each participating hospital (Kangdong Sacred Heart Hospital, Hallym University Sacred Heart Hospital, Kangnam Sacred Heart Hospital and Chuncheon Sacred Heart Hospital) for the review and publishing of information from the patients’ records. Informed consent was waived because of the retrospective nature of the study.

### Diagnosis of TBPE

TBPE was diagnosed based on the following criteria: (1) culture-positivity for MTB or positive TB-polymerase chain reaction (PCR) in pleural fluid or tissue; (2) granulomatous inflammation in pleural tissue; (3) pleural fluid combined with PTB resolved with anti-TB treatment; (4) lymphocytic exudates with high ADA levels (>40 U/L) in the pleural fluid, with cytological examination negative for malignancy and pleural effusion cleared by anti-TB treatment [5, 6].

### Thoracentesis and microbiologic examination

Fifty-milliliters of pleural fluid specimens was obtained during thoracentesis, collected in sterile polypropylene tubes, and transported to the laboratory. Of these, 5 mL of pleural fluid was concentrated, but not decontaminated, and inoculated for MTB culture as recommended [19]. AFB smears were prepared by auramine—rhodamine fluorescence staining and confirmed by Ziehl-Neelsen staining. MTB cultures were simultaneously performed with solid media, 3% Ogawa media (Eiken Chemical, Tokyo, Japan), and liquid media in the mycobacteria growth indicator tube 960 system (BD Biosciences, Franklin Lakes, NJ, USA).

### Statistical analysis

The data are presented as the median and interquartile range (IQR) for continuous variables and numbers (percentage) for categorical variables. The diagnostic consistency according to the number of pleural cultures was analyzed by McNemar’s test. To identify factors for predicting MTB growth in TBPE, clinical, radiological, and laboratory characteristics based on the positivity of the first and/or second pleural culture were compared by the Kruskal-Wallis test for continuous variables and the Chi-square test for categorical variables. The Wilcoxon test was performed to compare differences in pleural fluid biochemistry between the first and second thoracentesis. Multiple logistic regression analysis was performed to identify independent predictors of cultivation of MTB, as measured by the estimated odds ratios (OR) with 95% confidence intervals (CI), including variables with a *P*-value < 0.2 on univariate analysis. To reduce the risk of multicollinearity, one closely correlated variable was included as a candidate in the final model. All tests were two-sided and a P-value of less than 0.05 was considered statistically significant. Data were analyzed using IBM SPSS Statistics, version 24 (IBM, Armonk, NY, USA).

## Results

**Fig 1:**
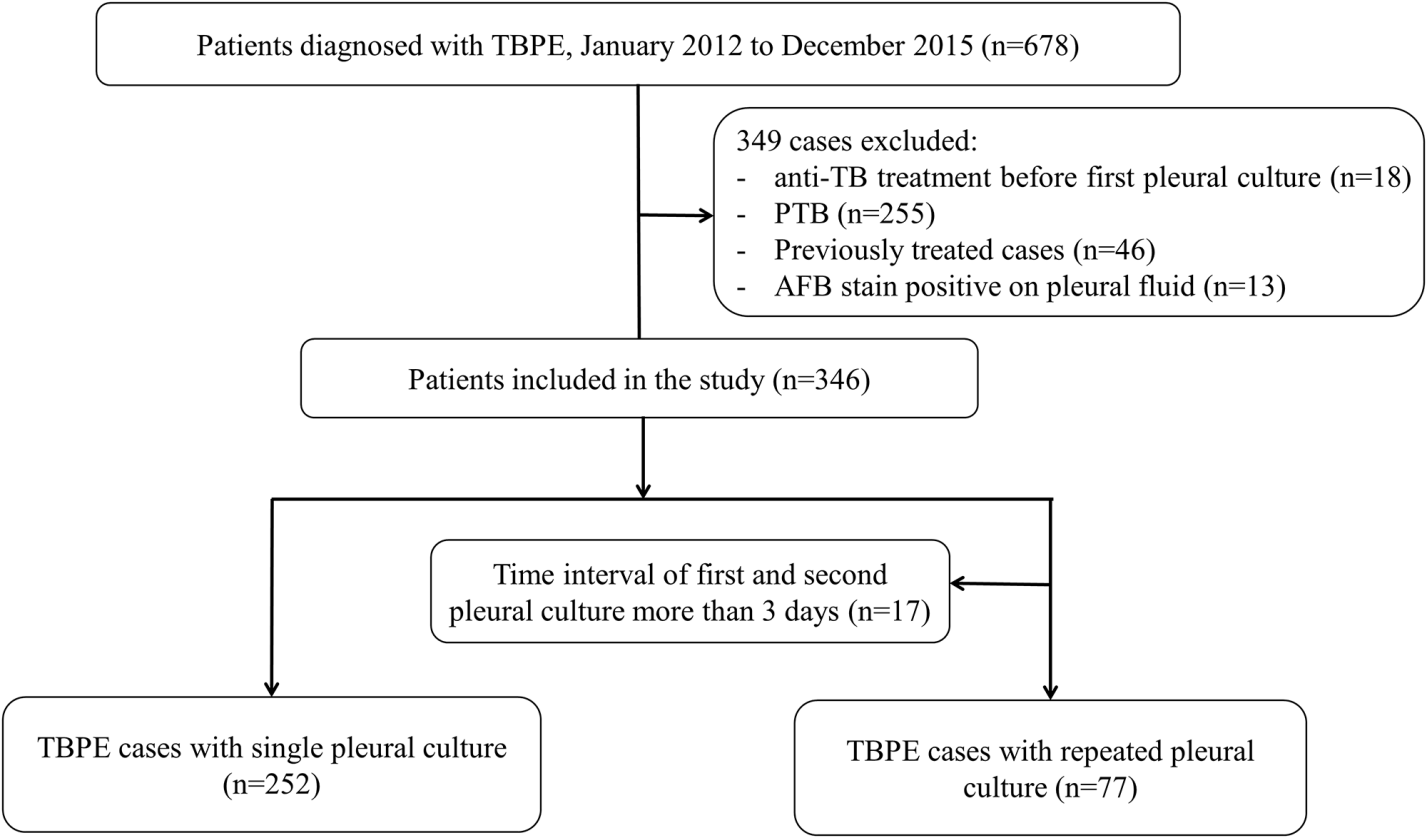
Flowchart of study: Selection of study participants.

Of the 4029 patients with TB from 2012 to 2015, 678 were diagnosed with TBPE. We excluded patients who received anti-TB treatment before the first pleural culture (n = 18), exhibited concurrent PTB (n = 255), had a history of treatment for TB (n = 46), or had a positive AFB smear for TBPE (n = 13) (Fig 1). In addition, we excluded 17 cases where the time interval between the first and second cultures was more than 3 days, as the goal of this study was to determine the diagnostic potential of the second culture [15–17].

A total of 329 TBPE cases were included and analyzed, including 109 culture-positive and 220 culture-negative cases for MTB in TBPE. Of the 220 patients who were culture-negative for MTB in TBPE, 14 were diagnosed by pleural biopsy, while 206 were diagnosed based on clinical criteria. Of the 329 total cases of TBPE, 252 cases provided a single pleural specimen for MTB culture, while the other 77 provided repeated pleural cultures.

The main focus of this study is the repeated-culture group, and the demographic and descriptive data of the 77 cases in this group are summarized in Table 1. There were 52 male subjects (67.5%) with a median age of 49.0 (IQR, 34.5–70.0) years. Among the 77 cases in the repeated-culture group, loculated TBPE was identified in 36 cases (46.8%) and the time interval between the first and second pleural cultures was a median of 1 day (1.0–2.0).

**Table 1:** Baseline characteristics of 329 patients with TBPE.

	Single culture	Repeated culture	*P*-value
	(n = 252)	(n = 77)	
Age, years	46.0 (29.3–66.8)	49.0 (34.5–70.0)	0.4
Male gender, %	138 (54.8)	52 (67.5)	0.049
Time interval between first and second culture	NA	1 (1.0–2.0)	NA
Comorbidity			
COPD and asthma	15 (6.0)	4 (5.2)	0.989
Chronic liver disease	3 (1.2)	1 (1.3)	0.999
Diabetes	28 (11.1)	13 (16.9)	0.235
Cerebrovascular disease	8 (3.2)	6 (7.8)	0.103
Cardiovascular disease	12 (4.8)	6 (7.8)	0.388
Hypertension	43(16.7)	19 (24.7)	0.182
CKD	6 (2.4)	5 (6.5)	0.138
Rheumatic disease	5 (2.0)	4 (5.2)	0.222
Malignancy*	0	6 (7.8)	<0.001
Radiological feature of TBPE			
Loculation	93 (36.9)	36 (46.8)	<0.001
Amount†			
Large	43 (17.1)	12 (15.6)	0.395
Moderate	176 (69.8))	59 (76.6)	
Small	33 (13.1)	6 (7.8)	
Effusion site			
Right	138 (54.8)	44 (57.1)	0.794
Left	114 (45.2)	33 (42.9)	
Pleural fluid			
Overall MTB culture-positive	75 (29.8)	34 (44.2)	<0.001
only in first culture		9 (11.7)	
only in second culture		10 (13.0)	
in both first and second culture		15 (19.5)	
MTB-PCR positive	3 (1.2)	5 (6.5)	0.019
SG	1.038 (1.036–1.040)	1.038 (1.034–1.040)	0.339
WBC (total cells)	2160.0 (1085.0–4120.0)	1860.0 (572.5–3425.0)	0.138
Lymphocyte, %	80.0 (64.3–90.0)	79.0 (58.0–90.0)	0.747
Neutrophil, %	10.0 (3.5–20.0)	6.0 (2.0–34.0)	0.985
pH	7.363 (7.313–7.411)	7.368 (7.282–7.413)	0.316
Glucose (mg/dL)	88.0 (73.0–111.5)	85.0 (66.5–107.5)	0.444
Protein (g/dL)	5.2 (4.7–5.6)	5.1 (4.5–5.5)	0.272
Albumin (g/dL)	2.9 (2.6–3.2)	2.8 (2.4–3.1)	0.12
LDH (IU/L)	523.5 (351.8–811.8)	587.0 (452.8–998.5)	0.13
ADA (IU/L)	86.7 (71.2–102.9)	84.4 (65.9–110.6)	0.494

Data are presented as the number of patients (%) or median (IQR)

TBPE, tuberculous pleural effusion; IQR, interquartile range; NA, not applicable; COPD, chronic obstructive pulmonary disease; CKD, chronic kidney disease; MTB, *Mycobacterium tuberculosis*; PCR, polymerase chain reaction; WBC, white blood cell; SG, specific gravity; LDH, lactate dehydrogenase; ADA, adenosine deaminase

*Prostate cancer (n = 1), ovary cancer (n = 1), bladder cancer (n = 1), rectal cancer (n = 1), gastric carcinoid (n = 1) and renal cell carcinoma (n = 1)

### Diagnostic yield from single and repeated pleural cultures for MTB

In the single-culture group, 29.8% (75/252) of the cases were culture-positive for MTB in TBPE. In the repeated-culture group, MTB was cultured from 31.2% (24/77) of the cases on the first pleural culture, which did not significantly differ from the results of the single-culture group (*P* = 0.887). However, the second pleural culture showed 13.0% (10/77) greater yield of MTB than did the first MTB culture trial. In the repeated-culture group, 44.2% (34/77) of cases eventually yielded a positive culture for MTB (Table 1). Finally, the diagnostic yield of MTB in TBPE was significantly increased by the second pleural culture in the repeated-culture group (31.1% vs. 44.2%, *P* < 0.001). Among the 34 MTBs isolated from the repeated-culture group, the DST suggested that pathogens were susceptible to all except four anti-TB drugs. Three isolates were resistant to INH only and one isolate was resistant to isoniazid and para-aminosalicylic acid.

**Fig 2:**
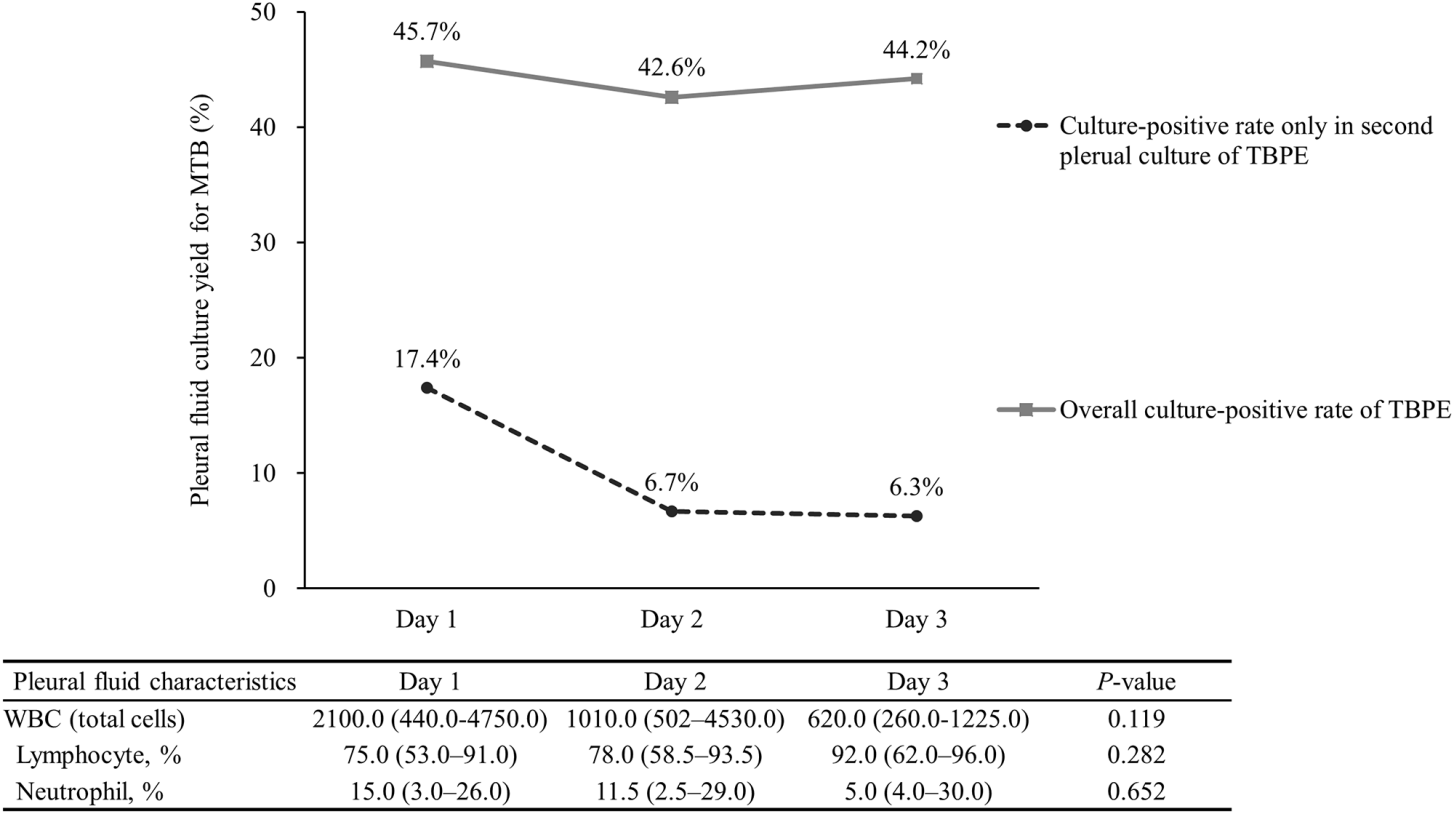
Positive culture rate of overall and second pleural culture according to time interval between first and second culture and changes in pleural fluid characteristics.

However, the increase in MTB yield via second pleural culture decreased over several days from 17.4% to 6.7% and eventually to 6.3% when the time interval between the first and second culture was increased (Fig 2). The median total white blood cell count and neutrophil percentage showed decreasing trends over time, but the lymphocyte percentage increased. However, the differences were not significant.

### Biochemical changes in TBPE in sequential examinations

Table 2 shows the biochemical changes in sequential examinations of the 77 cases in the repeated pleural culture group between the first and second pleural tappings. Even though lymphocyte predominance persisted, the proportion of neutrophils increased in culture-positives group only in the second culture or remained the same in culture-positive groups in both cultures. In contrast, lymphocyte predominance persisted and the proportion of neutrophils decreased in culture-positive group only in the first culture and culture-negative group in both cultures.

**Table 2:** Biochemical changes in sequential examinations of 77 cases in the repeated pleural culture group.

	First tapping	Second tapping	
Pleural culture fluid characteristics			*P*-value
Positive MTB in both pleural cultures (n = 15)			
WBC (total cells)	1320.0 (291.0–3750.0)	1730.0 (575–7720)	0.248
Lymphocyte, %	81.0 (54.8–93.5)	66.0 (46.3–94.0)	0.656
Neutrophil, %	7.5 (1.8–45.8)	19.0 (2.8–47.0)	0.799
Positive MTB only in first pleural culture (n = 9)			
WBC (total cells)	2120.0 (1775.0–4685.0)	795.0 (272.0–1890.0)	0.017
Lymphocyte, %	54.0 (16.5–71.0)	79.0 (48.8–89.5)	0.068
Neutrophil, %	35.0 (11.5–64.0)	10.5 (4.8–31.3)	0.063
Positive MTB only in second pleural cultures (n = 10)			
WBC (total cells)	460 (183.8–2165.0)	570.0 (277.5–2062.5)	0.735
Lymphocyte, %	81.5 (59.3–88.5)	80.0 (29.5–91.3)	0.326
Neutrophil, %	5.0 (1.0–30.0)	17.0 (5.0–47.3)	0.036
No MTB growth in both pleural culture (n = 43)			
WBC (total cells)	2130.0 (685.0–3450.0)	1225.0 (357.5–5267.5)	0.517
Lymphocyte, %	81.5 (60.8–92.3)	81.0 (65.80–95.8)	0.352
Neutrophil, %	5.5 (1.0–20.8)	4.5 (1.3–15.0)	0.619

Data are presented as median (IQR)

MTB, *Mycobacterium tuberculosis*; WBC, white blood cell

### Predictors for MTB-positive cultures of first and second pleural culture

Univariate comparisons of the clinical, radiological, and laboratory data of 77 TBPE patients with repeated pleural cultures are presented in Table 3. When samples were grouped into culture-positive from the first pleural culture, culture-positive only from the second pleural culture, and no growth from either pleural culture, there were no significant differences except for loculation, positive MTB-PCR, and lactate dehydrogenase (LDH) level.

**Table 3:** Comparisons of clinical, radiological, and laboratory characteristics based on MTB culture-positivity of serial pleural fluid in 77 TBPE patients.

	Positive in first culture	Positive only in second culture	No growth	*P*-value
	(n = 24)	(n = 10)	(n = 43)	
Age, years	49.0 (34.5–68.3)	45.5 (31.0–73.75)	51.0 (35.0–70.0)	0.893
Male gender, %	14 (58.3)	8 (80.0)	30 (69.8)	0.42
Time interval between first and second culture	1.0 (1.0–3.0)	1.0 (1.0–1.3)	1.0 (1.0–2.0)	0.34
Comorbidity				
COPD and asthma	1 (4.2)	0	3 (7.0)	0.645
Chronic liver disease	1 (4.2)	0	0	0.327
Diabetes	2 (8.3)	2 (20.0)	9 (20.9)	0.402
Cerebrovascular disease	2 (8.3)	0	4 (9.3)	0.609
Cardiovascular disease	2 (8.3)	1 (10.0)	3 (7.0)	0.943
Hypertension	6 (25.0)	4 (40.0)	9 (20.9)	0.452
CKD	2 (8.3)	1 (10.0)	2 (4.7)	0.75
Rheumatic disease*	1 (4.2)	2 (20.0)	1 (2.3)	0.073
Malignancy	2 (8.3)	1 (10.0)	3 (7.0)	0.943
Radiological features of TBPE				
Loculation	20 (83.3)	8 (80.0)	8 (18.6)	<0.001
Amount†				
Large	2 (8.3)	1 (10.0)	9 (20.9)	0.213
Moderate	18 (75.0)	9 (90.0)	32 (74.4)	
Small	4 (16.7)	0 (0.0)	2 (4.7)	
Effusion site				
Right	13 (54.2)	6 (60.0)	25 (58.1)	0.934
Left	11 (45.8)	4 (40.0)	18 (41.9)	
Pleural fluid, first thoracentesis				
MTB-PCR positive	4 (16.7)	1 (10.0)	0	0.026
SG	1.038 (1.035–1.040)	1.040 (1.026–1.042)	1.038 (1.034–1.040)	0.842
WBC (total cells)	1855.0 (590.0–3732.5)	460.0 (183.8–2165.0)	2130.0 (685.0–3450.0)	0.083
Lymphocyte, %	65.0 (50.0–90.0)	81.5 (59.3–88.5)	81.5 (60.8–92.3)	0.354
Neutrophil, %	20.0 (3.0–58.0)	5.0 (1.0–30.0)	5.5 (1.0–20.8)	0.099
pH	7.338 (7.224–7.394)	7.266 (7.227–7.392)	7.394 (7.301–7.428)	0.046
Glucose (mg/dL)	78.0 (54.0–106.8)	87.0 (46.0–134.5)	95.0 (74.0–108.0)	0.186
Protein (g/dL)	5.1 (4.7–5.4)	5.5 (3.0–5.7)	5.2 (4.5–5.4)	0.721
Albumin (g/dL)	2.9 (2.5–3.1)	2.8 (1.4–3.2)	2.8 (2.3–3.1)	0.786
LDH (IU/L)	778.5 (553.3–1185.0)	555.0 (398.5–1265.0)	529.0 (360.0–899.0)	0.02
ADA (IU/L)	93.4 (72.2–106.2)	84.3 (36.6–125.6)	81.6 (66.3–111.4)	0.853
Pleural fluid, second thoracentesis				
WBC (total cells)	1440.0 (467.0–3375.0)	570.0 (277.5–2062.5)	1225.0 (357.5–5267.5)	0.343
Lymphocyte, %	70.5 (47.0–93.3)	80.0 (29.5–91.3)	81.0 (65.80–95.8)	0.275
Neutrophil, %	17.5 (5.3–34.4)	17.0 (5.0–47.3)	4.5 (1.3–15.0)	0.047

Data are presented as the number of patients (%) or median (IQR)

TBPE, tuberculous pleural effusion; IQR, interquartile range; NA, not applicable; COPD, chronic obstructive pulmonary disease; CKD, chronic kidney disease; MTB, *Mycobacterium tuberculosis*; PCR, polymerase chain reaction; WBC, white blood cell; SG, specific gravity; LDH, lactate dehydrogenase; ADA, adenosine deaminase

*Gout (n = 3), Sjogren syndrome (n = 1) and rheumatoid arthritis (n = 1)

^†^The amount of TBPE was classified as small, moderate, or large based on the following findings on chest radiographs prior to pleural tapping: (1) small, a level of TBPE that blunted the costophrenic angle but did not obscure the entire diaphragm; (2) moderate, a level that obscured the entire diaphragm but was below the hilum; (3) large, a level up to and above the hilum.

To identify clinical predictors of MTB cultivation in TBPE after the first and second pleural culture, further multivariate logistic regression was conducted. In the first culture trial, the loculation of TBPE (adjusted OR, 14.967; 95% CI, 3.595–62.314; *P* < 0.001) and neutrophil percentage (adjusted OR, 1.031; 95% CI, 1.001–1.062; *P* < 0.001) were independently associated with culture-positivity for MTB in TBPE, after adjusting for potential confounding factors with P-values less than 0.2 in univariate analysis. In the second culture trial, the loculation of TBPE (adjusted OR, 5.799; 95% CI, 1.488–22.549; *P* = 0.011) and neutrophil percentage (adjusted OR, 1.039; 95% CI, 1.003–1.076; *P* < 0.001) were independently associated with the cultivation of MTB in the same manner (Table 4).

**Table 4:** Predictors of MTB cultivation in patients with tuberculous pleural effusion via first and second pleural culture.

	Univariate analyses		Multivariate logistic regression analyses	
	OR (95% CI)	*P* value	Adjusted OR (95% CI)	*P* value
**First pleural culture for MTB isolation**				
Rheumatic disease	10.932 (0.244−489.436)	0.218		
Loculated TBPE	17.123 (3.501−83.745)	<0.001	14.061 (3.503–56.447)	<0.001
Pleural fluid				
MTB-PCR positive	13.677 (0.286−654.824)	0.083		
Lymphocyte, %	1.061 (0.969−1.162)	0.203		
Neutrophil, %	1.114 (0.991−1.251)	0.070	1.031 (1.001–1.062)	0.041
pH	0.084 (0.012−0.064)	0.185		
Glucose (mg/dL)	0.993 (0.967−1.020)	0.600		
LDH (IU/L)	1.000 (0.998−1.002)	0.980		
**Second pleural culture for MTB isolation**				
Rheumatic disease	0.251 (0.020−3.138)	0.283		
Loculated TBPE	6.032 (1.514−26.239)	0.011	5.799 (1.488–22.549)	0.011
Pleural fluid				
Lymphocyte, %	1.061 (0.969−1.162)	0.202		
Neutrophil, %	1.117 (0.993−1.257)	0.064	1.039 (1.003–1.076)	0.034
Toward lymphocyte predominance	0.866 (0.184−4.082)	0.856		
Toward neutrophil predominance	0.572 (0.130−2.521)	0.308		

TBPE, tuberculous pleural effusion; MTB, *Mycobacterium tuberculosis*; PCR, polymerase chain reaction; LDH, lactate dehydrogenase

## Discussion

In this study, we evaluated whether repeated pleural cultures are associated with increased MTB growth from pure TBPE, excluding cases with combined PTB or positive AFB smear for TBPE. This study revealed important findings regarding the culture of MTB in TBPE. First, the yield of MTB from TBPE patients increased by approximately 13% if pleural cultures were repeated (Table 1). Second, the additional isolation of MTB via second pleural culture decreased over time. Thus, the effect of second pleural culture may be maximized when it is conducted for 2 days in a row. These results may be related to the insufficient immune clearance for invading of MTB into the pleural space (Table 2 and Fig 2). This result can be applied for MTB isolation in TBPE and a continuous spectrum of disease processes. Moreover, the loculation and neutrophil percentage of TBPE were positively associated with the probability of MTB culture from TBPE (Table 4). Thus, the association between additional culture-positivity for MTB and biochemical changes of TBPE was determined in this study. This is the first study to evaluate the diagnostic value of additional pleural cultures for identifying MTB.

Unlike the traditional concept of pathogenesis of TBPE, which involves a delayed hypersensitivity response of TB protein, it is now considered a spectrum, with the occurrence of direct MTB infection into the pleural space followed by serial immunological reactions to clear the pathogen [5, 20]. Thus, the possibility of MTB cultivation is increased if there is insufficient immunological clearance. We observed interesting results when we compared the biochemical characteristics of TBPE. In cases where both pleural cultures were positive, the proportion of neutrophils remained constant over time even though lymphocyte predominance persisted. Moreover, the proportion of neutrophils increased culture-positives group only in the second culture. In contrast, lymphocyte predominance continued, and the proportion of neutrophils decreased in cases where both pleural cultures were negative, as well as cases where only the first pleural culture was positive.

This new concept of TBPE pathogenesis allows for the identification of predictors of MTB cultivation. Previous studies have reported that low lymphocyte percentage, high neutrophil percentage, low glucose and pH levels, and high LDH level of TBPE, which suggest ongoing inflammation, are associated with MTB culture in TBPE [4, 13, 21, 22]. Moreover, loculation of TBPE is well-known as an intense intra-pleural inflammation and clinical predictor of MTB culture [23–25]. Similarly, loculation and a high neutrophil percentage were independently associated with culture-positivity for MTB in TBPE in our study.

Conventionally, pleural biopsy and culture of tissue are considered the most accurate modalities for diagnosis of TB pleuritis. Despite advances in ultrasonography-guided and thoracoscopic pleural biopsy, this method remains invasive with fatal complication risks [5, 6, 26]. Moreover, in TB endemic areas including South Korea, TB pleurits is conducted according to clinical, radiological, and laboratory findings, except for complex cases.

Thus, several studies have been carried out to develop a more effective method for isolating MTB from TBPE [4, 12–14]. Previous studies have shown that liquid media culture and MODS culture resulted in greater MTB growth as compared to solid media culture [11, 12]. Moreover, MTB identification was improved when a combination of solid and liquid media was used as compared to using only one type of media [13, 27]. However, additional methods are needed to increase the growth of MTB because much of the TBPE remains in the gray zone. Interestingly, a recent study showed that a larger volume culture of TBPE improved the growth of MTB, but this was less effective than expected [14]. Therefore, we attempted to develop other methods for increasing culture yield.

In the diagnosis of PTB, the WHO recommends repeating more than two sputum cultures for MTB culture, based on the diagnostic yield of consecutive microbiological exams [15]. On this basis, we presumed that repeated pleural cultures would also improve the growth of MTB. We excluded cases with combined PTB and TBPE or positive AFB smear for TBPE, because MTB as a pathogen is easy to isolate in these cases and does not typically require additional effort such as repeated pleural culture for MTB [4, 28]. The goal of this study was to develop a method for isolating MTB in only TBPE within the grey-zone of culture-negativity for MTB. Therefore, repeating the pleural culture of TBPE was expected to improve MTB growth in clinical practice. The diagnostic yield of this method should be evaluated particularly in specimens from patients with TBPE without PTB and positive AFB smear for TBPE [29]. In this study of patients with TBPE only, repeated pleural cultures showed 13% (10/77) greater MTB growth overall. Furthermore, the yield of second cultures decreased over time from 17.4% to 6.7% to eventually 6.3%, similar to that in previous sputum studies [16].

In this study, we conducted repeated pleural cultures over a 3-day period because this was a retrospective observational study. In South Korea, an intermediate TB-burden country, clinicians prescribe anti-TB medication after identifying pleural fluid characteristics such as lymphocytic exudates with high ADA levels (>40 U/L) before microbiological confirmation of MTB, which is based on the Korea Guidelines for Tuberculosis. In academic hospitals, pleural fluid characteristics, including pleural CEA for differentiating from malignancy, are reported within 1 day. However, cytological examination results are typically reported within 2–3 days. Thus, most TBPE cases are administered anti-TB medication on the same day of thoracentesis, the first pleural culture for MTB. Moreover, TBPE is a paucibacillary disease and therefore, accuracy can be decreased and the results will be less informative if the time interval between the first and second pleural cultures is extended. For PTB, repeated sputum exams using specimens obtained over 3 consecutive days have been recommended in many guidelines. Thus, we limited the time frame of the second culture to a 3-day period.

There were several limitations to this study. First, the results of this study were based on retrospective data and a relatively small sample size in the repeated-culture group, despite being conducted in multiple centers. Thus, we cannot definitively conclude that the repeated pleural culture increases the isolation of MTB by up to 13.0% in all TBPE cases. However, because the goal of this study was to improve the diagnostic yield from cases of TBPE alone, we classified MTB culture-positive respiratory specimens as PTB regardless of evidence of radiological involvement of lung parenchyma, unlike in a previous study [29]. As described in the introduction, the aim of this study was to develop a method for reducing the grey-zone in TBPE diagnosis through microbiological confirmation. Therefore, we excluded 24 TBPE cases with MTB culture-positive respiratory specimens, although these subjects had provided a second pleural culture and had no radiological evidence of PTB.

Second, clinical decisions to collect second pleural cultures were based on the clinical features of TBPE patients and the actual yield of MTB growth may be biased. Since 2012, some pulmonologists in our medical centers have conducted inter-costal drainage by inserting a pig-tail tube in suspected TBPE cases when possible. This was carried out either for effective drainage or reduction of residual pleural thickening with or without fibrinolysis therapy, particularly in cases with loculated TBPE. Moreover, we conducted additional pleural fluid culture for MTB isolation for 2 consecutive days. Repeated pleural cultures were performed on different days. This protocol was applied for most cases (69/77, 89.6%) in this study. In the remaining 8 cases of TBPE, additional pleural culture was conducted during repeated thoracentesis for symptom relief.

Third, this study showed a relatively low rate of culture-positive TBPE compared to the rate outside South Korea (109/329, 33.1% vs. 50.0% to 63.1%) [4, 14]. However, most studies in Korea showed a similar culture-positive rate (15.0–41.3%). It should be noted that some of the other studies included cases of TBPE with PTB [4, 13, 14, 23, 30]. Fourth, this study was performed in an area with an intermediate TB rate and low human immunodeficiency virus (HIV) infection burden. There were no HIV-infected cases enrolled in our study or included in the analysis. Thus, our results may not be generalized to other clinical situations, and further studies are needed in order to determine whether these results can be applied.

## Conclusions

In conclusion, repeated pleural cultures yielded increased cultures of MTB from TBPE in HIV-negative individuals and were particularly effective when conducted for 2 consecutive days. This suggests that repeated pleural cultures are advantageous for isolating MTB from specimens of patients with TBPE without PTB and positive AFB smear for TBPE, for which no other simple methods for culture growth can be used.

## Supporting information

S1 File Raw data. (XLSX)

## Abbreviations

ADA: adenosine deaminase
AFB: acid-fast bacilli
CKD: chronic kidney disease
COPD: chronic obstructive pulmonary disease
DR: drug-resistant
DST: drug sensitivity test
EPTB: extrapulmonary tuberculosis
HIV: human immunodeficiency virus
IQR: interquartile range
LDH: lactate dehydrogenase
MTB: *Mycobacterium tuberculosis*
NA: not applicable
PCR: polymerase chain reaction
PTB: pulmonary tuberculosis
SG: specific gravity
TB: tuberculosis
TBPE: tuberculous pleural effusion
WBC: white blood cell
WHO: World Health Organization
